# Investigating Efficient Risk-Stratified Pathways for the Early Detection of Clinically Significant Prostate Cancer

**DOI:** 10.3390/jpm14020130

**Published:** 2024-01-23

**Authors:** Juan Morote, Ángel Borque-Fernando, Luis M. Esteban, Ana Celma, Miriam Campistol, Berta Miró, Olga Méndez, Enrique Trilla

**Affiliations:** 1 Department of Urology, Vall d’Hebron Hospital, 08035 Barcelona, Spain; ana.celma@vallhebron.cat (A.C.); miriam.campistol@vallhebron.cat (M.C.); enrique.trilla@vallhebron.cat (E.T.); 2 Department of Surgery, Universitat Autònoma de Barcelona, 08193 Bellaterra, Spain; 3 Research Group in Urology, Vall d’Hebron Research Institute, 08035 Barcelona, Spain; olga.menzez@vhir.org; 4 Department of Urology, Hospital Miguel Servet, IIS-Aragon, 50009 Zaragoza, Spain; aborque@comz.org; 5 Department of Applied Mathematics, Escuela Universitaria Politécnica La Almunia, Universidad de Zaragoza, 50100 Zaragoza, Spain; lmeste@unizar.es; 6 Statistic Unit, Vall d’Hebron Research Institute, 08035 Barcelona, Spain; berta.miro@vhir.org

**Keywords:** screening, prostate cancer, Barcelona risk calculator, Proclarix, risk-stratified pathway

## Abstract

Risk-stratified pathways (RSPs) are recommended by the European Association of Uro-logy (EAU) to improve the early detection of clinically significant prostate cancer (csPCa). RSPs can reduce magnetic resonance imaging (MRI) demand, prostate biopsies, and the over-detection of insignificant PCa (iPCa). Our goal is to analyze the efficacy and cost-effectiveness of several RSPs by using sequential stratifications from the serum prostate-specific antigen level and digital rectal examination, the Barcelona risk calculators (BCN-RCs), MRI, and Proclarix™. In a cohort of 567 men with a serum PSA level above 3.0 ng/mL who underwent multiparametric MRI (mpMRI) and targeted and/or systematic biopsies, the risk of csPCa was retrospectively assessed using Proclarix™ and BCN-RCs 1 and 2. Six RSPs were compared with those recommended by the EAU that, stratifying men from MRI, avoided 16.7% of prostate biopsies with a prostate imaging–reporting and data system score of <3, with 2.6% of csPCa cases remaining undetected. The most effective RSP avoided mpMRI exams in men with a serum PSA level of >10 ng/mL and suspicious DRE, following stratifications from BCN-RC 1, mpMRI, and Proclarix™. The demand for mpMRI decreased by 19.9%, prostate biopsies by 19.8%, and over-detection of iPCa by 22.7%, while 2.6% of csPCa remained undetected as in the recommended RSP. Cost-effectiveness remained when the Proclarix™ price was assumed to be below EUR 200.

## 1. Introduction

Thirty years after the U.S. Food and Drug Administration (FDA) approved serum prostate-specific antigen (PSA) testing for the early detection of prostate cancer (PCa) [1,2], the European Union (EU) recommended screening for PCa. The EU currently encourages member states to assess the feasibility and effectiveness of a risk-organized PCa screening by utilizing PSA testing and magnetic resonance imaging (MRI) scanning as a follow-up measure [3,4,5,6]. The currently recommended screening focuses on the early detection of clinically significant PCa (csPCa), aiming to avoid an excessive number of unnecessary prostate biopsies and the over-detection of insignificant PCa (iPCa), which were highly criticized in the past [7]. The last European Council meeting on cancer screening, in March 2022, emphasized the importance of a stepwise approach involving pilot programs and further research to assess the feasibility and effectiveness of risk-organized PCa screening programs [8]. These pilot programs will invite men at risk of PCa and will apply individual retesting intervals, using PSA testing for initial screening and follow-up tests with risk calculators and magnetic resonance imaging before prostate biopsy [5]. As part of this effort, the EAU PRAISE-U (Prostate Cancer Awareness and Initiative for Screening in the European Union) project was selected by the European Commission to receive funding under the EU4Health program [9]. Risk-stratified pathways (RSPs) are needed for the efficient development of csPCa screening. These pathways should incorporate efficient tools for sequencing csPCa risk stratifications in men suspected of having PCa, with the goals of minimizing MRI scanning demand and unnecessary prostate biopsies and mitigating the over-detection of iPCa [4,5,6].

PSA density, modern tumor markers, and predictive models are the most recommended tools to stratify men suspected of having PCa [10]. PSA density contributes to discriminating csPCa, requiring an accurate measurement of prostate volume. Currently, PSA density is often used after the prostate volume derived from an MRI has been esta-blished [11]. Most MRI-based predictive models incorporate calculated PSA density, serum PSA levels, and MRI-derived prostate volume [12]. Modern tumor markers are efficient tools for stratifying men suspected of having PCa before and after an MRI. However, their drawbacks include the need for new blood or urine sample procurement for asses-sment of PCa and associated costs [13,14,15,16,17,18]. While predictive models are efficient tools, the design of user-friendly web or smartphone applications is crucial for easy and quick utilization. Currently, predictive models for csPCa are available both before and after MRI; however, validations at the specific sites of future use are essential [19,20,21,22].

Proclarix™ is a novel tumor marker developed for csPCa detection that incorporates the serum measurements of thrombospondin 1 (THBS1), cathepsin D (CTSD), total PSA, and percent free PSA, along with age. Proclarix™ has been shown to be effective in redu-cing unnecessary MRI scans in men suspected of having PCa [23], as well as prostate biopsies before MRI, especially in those men with a prostate imaging–reporting and data system (PI-RADS) score of three or less [24]. The Barcelona predictive models (BCN-PMs) have been developed and externally validated for predicting the risk of csPCa in prostate biopsies before and after MRI, with corresponding web risk calculators (BCN-RC 1 and BCN-RC2) [20,22]. BCN-RC 1, designed to predict csPCa before MRI scanning, incorporates age, PCa family history, serum PSA, DRE characteristics, and the DRE-prostate vo-lume category [20]. The DRE prostate volume category was incorporated since prostate volume, which is an important predictor of PCa, is not usually available just before PCa suspicion [25]. BCN-RC 2 incorporates the same variables as BCN-RC 1 but substitutes the DRE prostate volume category with the true prostate volume assessed from MRI and PI-RADS score [22]. It has also been observed that men suspected of having PCa with serum PSA levels exceeding 10 ng/mL and a suspicious digital rectal examination (DRE) can undergo systematic biopsies without the risk of misdiagnosing csPCa [26]. Recently, some RSPs for csPCa detection stratified men suspected of having PCa from serum PSA and DRE in the first stratification, followed by a second stratification from BCN-RC 1, and a third one from BCN-RC 2, and have been reported with good results [27]. Stratifications of men suspected of having PCa from the Rotterdam RC3 and the Rotterdam MRI-RC have also been reported in the PRECISION trial [28]. Recently, the study IP1-PROSTAGRAM proposed several RSPs using different cut-off points for serum PSA and the PI-RADS score for stratifications of men suspected of having PCa [29].

The methodology to compare RSPs and assess their effectiveness is difficult. However, risk groups, certain scores, and risk maps have been proposed in which only one outcome variable needs to be predicted [30,31,32,33]. Comparisons between RSPs for csPCa detection are more difficult since multiple endpoint variables should be computed as a decrease in MRI demand, prostate biopsies, and iPCa over-detection. For this reason, cost-effectiveness analysis can be useful for comparing the overall benefits of several RSPs [34].

Our aim is to investigate the efficacy and cost-effectiveness of some new RSPs. The proposed RSPs are based on sequential stratifications based on serum PSA levels and DRE characteristics of men suspected of having PCa, BCN-RC 1 and BCN-RC 2, multiparame-tric MRI (mpMRI), and Proclarix™.

## 2. Materials and Methods

### 2.1. Design, Setting, and Participants

A retrospective study was conducted involving 567 men suspected of having PCa, identified based on a serum PSA level greater than 3.0 ng/mL. These individuals underwent pre-biopsy mpMRI, followed by targeted and/or systematic biopsies. Targeted and systematic biopsies were performed when the PI-RADS score was >3, while systematic biopsies were conducted when the score was less than 3. This study was conducted from January 2018 to March 2020 at a single academic institution. The exclusion criteria comprised men with PCa on active surveillance and men receiving 5-⍺-reductase inhibitors. Approval for the present study was obtained from the institutional Ethics Committee (PR-AG129/2020 and PR-AG02/2021), and financial support was provided by the Instituto de Salut Carlos III (SP) and the European Union (PI20/01666).

### 2.2. Intervention

Proclarix™ assessments were conducted at Proteomedix Inc. in Zurich-Schlieren, Switzerland, for all participants. Serum samples were obtained just before prostate biopsy and stored at −80 °C (Ref. collection: 0003439, Biobancs.isiii.es). Individual risks of csPCa were determined by using BCN-RC 1 and BCN-RC 2, accessed through the web app at https://mripcaprediction.shinyapps.io/MRIPCaPrediction/, accessed on 22 November 2023.

### 2.3. MpMRI Technique and Evaluation

MRI was performed on a 3-Tesla scanner, utilizing a surface phased-array coil (Magneton Trio™, Siemens Corp., Erlangen, Germany). The acquisition protocol adhered to the European Society of Urogenital Radiology guidelines and included T2-weighted imaging (T2W), diffusion-weighted imaging (DWI), and dynamic contrast-enhanced (DCE) imaging. Two expert radiologists interpreted mpMRI scans in accordance with PI-RADS version 2.0.

### 2.4. Prostate Biopsy Procedure and Pathologic Analysis

Targeted biopsies were conducted, extracting 2–3 cores from each PI-RADS greater than 3 lesions, employing the transrectal MRI-TRUS cognitive fusion technique. Finally, 12-core systematic biopsies were obtained in all individuals. The biopsies were performed by a skilled urologist using a BK Focus 400™ ultrasound scanner (BK Medical Inc., Herlev, Denmark). Biopsy samples were sent individually to the pathology department, where two expert uropathologists analyzed them. Tumor grading was based on the International Society of Urologic Pathology (ISUP) grade group (GG), and csPCa was defined when GG was 2 or higher.

### 2.5. Proclarix™ Assessment

THBS-1, CTD, total PSA, and free PSA were measured by using specific immunoassays. Subsequently, an algorithm was employed to compute THBS-1 and CTD levels, along with total PSA, percent free PSA, and age. The outcome of this algorithm provided a risk score for csPCa, reported on a scale from 0 to 100%.

### 2.6. BCN-RC 1 and BCN-RC 2 Risk of csPCa Assessment

The individual risks of csPCa were evaluated based on various factors in BCN-RC 1 and BCN-RC 2. In BCN-RC 1, the assessment considered age (years), serum PSA level (ng/mL), DRE (normal vs. suspicious), type of biopsy (initial vs. repeated), family history of PCa (no vs. yes), and DRE prostate volume category (small, intermediate, large). The risk of csPCa from BCN-RC 2 incorporated additional parameters, including the PI-RADS score (1 to 5) and the prostate volume derived from MRI. The reported risks were scored on a scale from 0 to 100%.

### 2.7. Proposed Risk-Stratified Pathways for Analysis

We propose to analyze seven RSPs.

(1) The first RSP represents the commonly recommended pathway and serves as the control in our study. In this pathway, all individuals suspected of having PCa undergo mpMRI, and subsequent stratification is based on the PI-RADS score. A prostate biopsy is avoided if the PI-RADS score is less than 3. For men with a PI-RADS score greater than 3, targeted and systematic biopsies are conducted.
(2) The second RSP involves an initial stratification of men based on their serum PSA level and DRE characteristics (normal vs. suspicious). In this pathway, men with a PSA level exceeding 10 ng/mL and suspicious DRE skip mpMRI and proceed directly to systematic biopsies. On the other hand, men with a serum PSA level of 10 or lower or a normal DRE undergo mpMRI. Prostate biopsies are avoided for those with a PI-RADS score of less than 3, while targeted and systematic biopsies are performed for those with a PI-RADS score greater than 3.
(3) In the third RSP, men are initially stratified based on their serum PSA level and DRE characteristics, similar to the first RSP. The second stratification is then conducted using Proclarix™ for men with a serum PSA level less than 10 ng/mL or a normal DRE. In this pathway, individuals with Proclarix™ scores of 10% or less undergo follow-up, while those with scores exceeding 10% proceed to mpMRI and targeted and/or systematic biopsies in cases where the PI-RADS score is less than 3.
(4) The fourth RSP stratifies men based on serum PSA levels and DRE, similar to the first RSP. The second stratification is carried out using BCN-RC 1 in men with serum PSA levels of 10 or lower ng/mL or normal DRE. The chosen threshold of 12% was determined to maintain sensitivity, resulting in the same misdiagnosis rate of csPCa as the control pathway. Men with a csPCa risk of 12% or less undergo follow-up, while those with a risk greater than 12% undergo mpMRI, leading to a third stratification from BCN-RC 2. In this stage, a threshold of 0.7% was selected due to its 100% sensitivity in detecting csPCa. Consequently, men with a csPCa risk of 0.7 or less undergo follow-up, whereas those with a risk greater than 0.7% undergo targeted and/or systematic biopsies.
(5) The fifth RSP incorporates the initial two stratifications from PSA-DRE and Procla-rix™. Men with Proclarix™ scores of 10% or greater undergo mpMRI, and a subsequent third stratification from BCN-RC 2 is performed. In this instance, the threshold with 100% sensitivity for csPCa was determined to be 0.6%. Accordingly, individuals with a risk of 0.6% or less undergo follow-up, while those with a risk exceeding 0.6% undergo targeted and/or systematic biopsy.
(6) The sixth RSP stratifies men based on PSA-DRE and BCN-RC 1. Once the mpMRI is conducted, the third stratification is executed using Proclarix™. The threshold of Proclarix™ ensuring 100% sensitivity for csPCa in this context was determined to be 2%. Consequently, individuals with a csPCa risk of 2% or less undergo follow-up, while those with a risk exceeding 2% undergo targeted and/or systematic biopsy.
(7) Finally, the seventh RSP consists of four stratifications. The initial two are determined by PSA-DRE and BCN-RC 1. The third stratification is contingent upon the mpMRI results, where individuals with a PI-RADS greater than 3 undergo targeted and systematic biopsies, whereas those with a PI-RADS score of less than 3 are stratified based on Proclarix™. Men with Proclarix™ scores of 10% or less are scheduled for follow-up, while those with scores of 10% or higher undergo targeted and/or systematic biopsies.

### 2.8. Endpoint Variables

The clinical efficacy of REPs was assessed based on several key parameters, including the rates of avoided mpMRI scans and prostate biopsies, as well as the rates of undetected csPCa and the reduction in rates of over-detection of iPCa.

### 2.9. Statistical Analysis

Quantitative variables were expressed as median and interquartile range (IQR) va-lues. Qualitative variables were expressed as percentages. The number of csPCa and non-csPCa cases needed for finding a 10% difference in prostate biopsies as significant at the 0.05 level with 80% potency, from the 17.6% decrease observed in the recommended RSP, for a recruitment of 1:1.5 (case controls), corresponded to 228 csPCa and 342 non-csPCa (570 cases). The discrimination ability for csPCa of mpMRI, Proclarix™, BCN-RC 1, and BCN-RC 2 was analyzed through receiver operating characteristic (ROC) curves and areas under the curve (AUCs). Odds ratios and their 95% confidence intervals (95% CI) were calculated. Individual percent probabilities for csPCa from BCN-RC1 and BCN-RC2 were estimated through logistic regression. The results of Proclarix™ expressed the individual percent probability of csPCa, while the PI-RADS score (1 to 5) expressed a semiquantitative risk of csPCa [35]. Nonparametric estimations of the AUCs were calculated directly from the raw data using the Wilcoxon–Mann–Whitney two-sample statistic. The standard error of the AUCs was calculated by using DeLong et al.’s method [36]. RSPs were constructed from sequential stratifications based on PSA serum levels and DRE characteristics: BCN-RC 1, BCN-RC 2, Proclarix™, and mpMRI. The efficacy of RSPs was considered based on the reduction in MRI demand, prostate biopsies, and the over-detection of iPCa, when the csPCa misdiagnosis was fixed in 2.6% of cases, as established by the recommended RSP. Rates of avoided prostate biopsies were compared with the chi-square test. The cost-effectiveness analysis was based on the savings of RSPs estimated according to the reduction in MRI and prostate biopsies. Reference prices from Vall d´Hebron Hospital were applied. Cost-effectiveness was estimated according to a hypothetical Proclarix™ price. SPSS v.25 (IBM Corp., Armonk, NY, USA) and R programming language v.3.3.1 (The R Statistical Foundation, Vienna, Austria) were used.

## 3. Results

### 3.1. Characteristics of the Study Cohort

The median age of our study cohort of 567 men with a serum PSA level higher than 3.0 ng/mL was 69 years (63–74), with the median serum PSA level being 7.0 ng/mL (4.9–11.2). These men presented abnormal DRE in 19.2%. The percentage of men with previous negative prostate biopsies was 19.2%, and that of those with a family history of PCa was 23.5%. The PI-RADS score distribution corresponded to <3 in 17.6% of men, 3 in 29.8%, 4 in 33.5%, and 5 in 19.0%. Overall, PCa was detected in 296 men (52.2%), csPCa in 230 (40.6%), and iPCa in 66 (11.6%), as seen in Table 1.

### 3.2. Discrimination Ability for csPCa of Tools Used for Stratifications of Men Suspected of Having PCa

**Figure 1:**
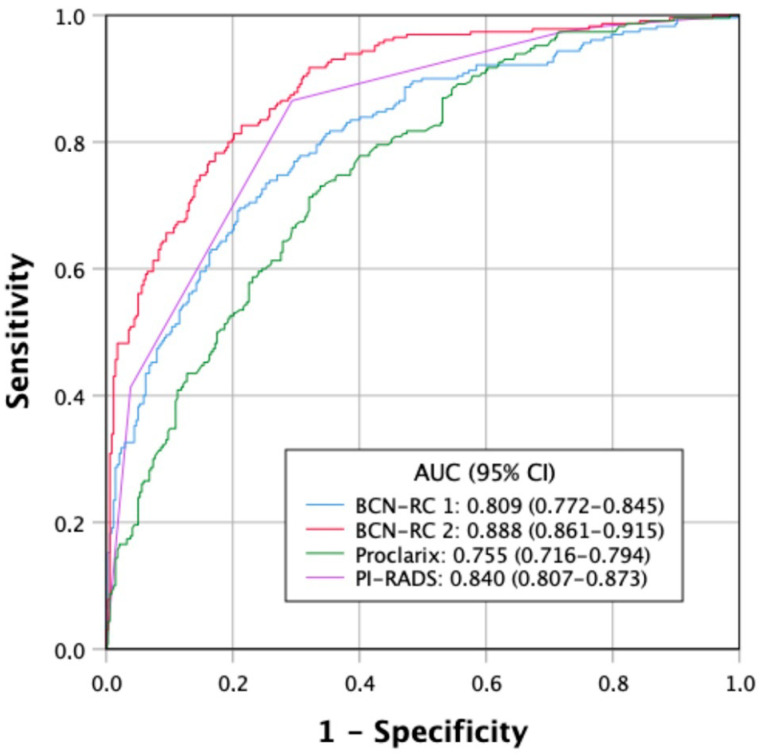
Receiving operating characteristic curves illustrating the discriminative ability for csPCa of the PI-RADS score, BCN-RC 1, BCN-RC 2, and Proclarix™.

Of 48 men with a serum PSA level higher than 10 ng/mL (8.5%) and suspicious DRE, csPCa was detected in 43 (89.6%), while of 519 men with a serum PSA level of 10 ng/mL or lower and normal DRE, csPCa was detected in 66 (12.7%), *p* < 0.001. ROC curves of the discrimination ability for csPCa of BCN-RC1, BCN-RC 2, Proclarix™, and PI-RADS are presented in Figure 1. The AUCs were 0.809 (95% CI 0.772–0.845) for BCN-RC 1, 0.888 (0.861–0.915) for BCN-RC 2, 0.755 (0.716–0.794) for Proclarix™, and 0.840 (0.807–0.873) for PI-RADS score, *p* < 0.001.

### 3.3. Behavior and Clinical Effectiveness of Proposed RSPs

**Figure 2:**
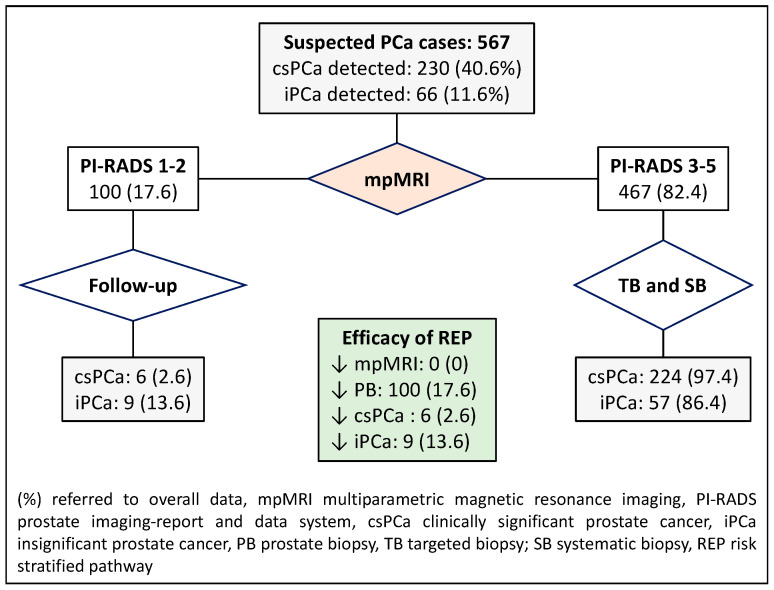
Flowchart and efficacy of the currently recommended RSP-1, which stratifies men suspected of having PCa according to the PI-RADS score. Green are overall efficacy.

Compared to the control pathway in which all men suspected of having PCa underwent mpMRI and targeted and/or systematic biopsies, the first RSP, based on the PI-RADS stratification, showed six cases with misdiagnoses of csPCa (2.6%) among 100 men (17.6%) with a PI-RADS score of less than 3 who would undergo follow-up. The 467 individuals (82.4%) with a PI-RADS score of 3 or higher would undergo targeted and systematic biopsies. This RSP would reduce prostate biopsies in 100 cases (17.6%) and iPCa over-detection in 9 (13.6%), Figure 2.

**Figure 3:**
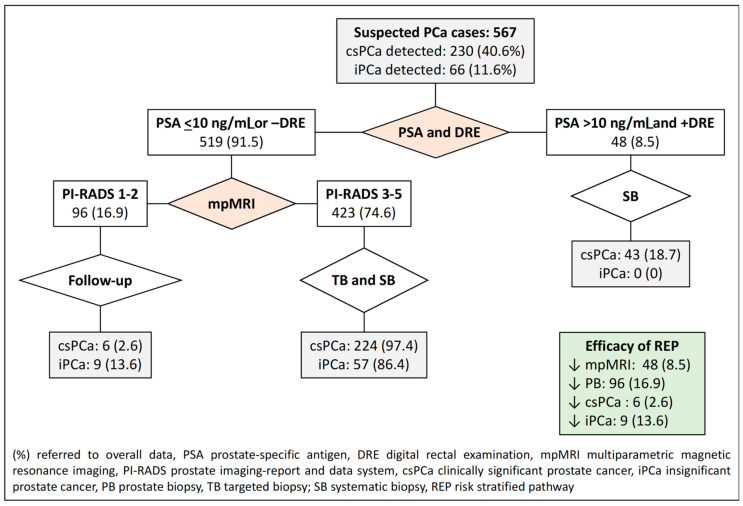
Flowchart and efficacy of the currently recommended RSP-2, which stratifies men suspected of having PCa according to the PI-RADS score. Green are overall efficacy.

The second RSP, based on PSA-DRE and PI-RADS stratifications, reduced MRI scans in 48 cases (8.5%), prostate biopsies in 96 (16.9%) when the PI-RADS score was less than 3, and iPCa over-detection in 9 (13.6%). Misdiagnosis of csPCa remained in six men (2.6%), as shown in Figure 3.

**Figure 4:**
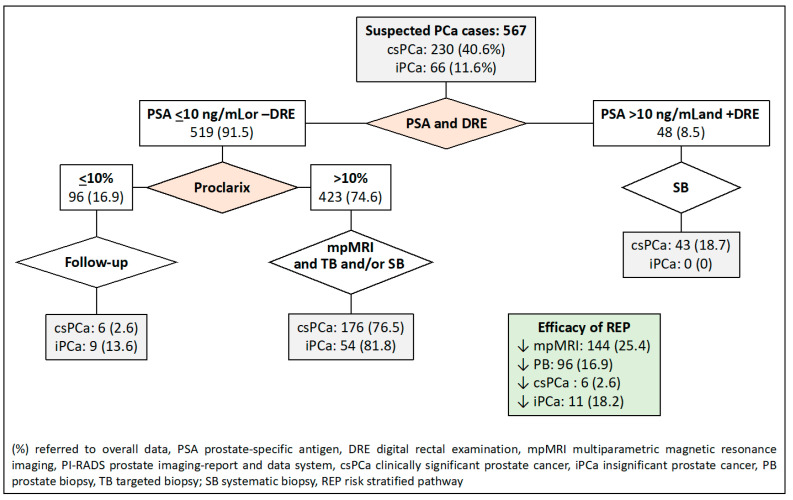
Flowchart and efficacy of the RSP-3, based on sequential stratifications from PSA-DRE, DRE, and Proclarix™. Green is overall results.

The third RSP, based on PSA-DRE and Proclarix™ stratifications, reduced MRI scans in 144 cases (25.4%), prostate biopsies in 96 cases (16.9%), and iPCa over-detection in 11 cases (18.2%), and csPCa remained undetected in 6 cases (2.6%), as shown in Figure 4.

**Figure 5:**
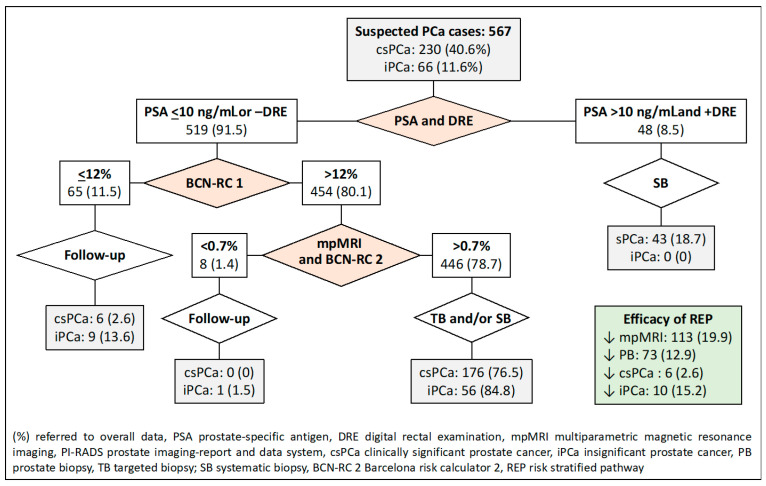
Flowchart and efficacy of the RSP-4, based on sequential stratifications from PSA-DRE, BCN-RC 1, and BCN-RC 2. Green is overall results.

The fourth RSP, based on PSA-DRE, BCN-RC 1, and BCN-RC 2 stratifications, reduced the demand for MRI scans in 113 individuals (19.9%), prostate biopsies in 73 (12.9%), and the over-detection of iPCa in 10 (15.2%). Misdiagnosis of csPCa remained in six cases (2.6%), as shown in Figure 5.

**Figure 6:**
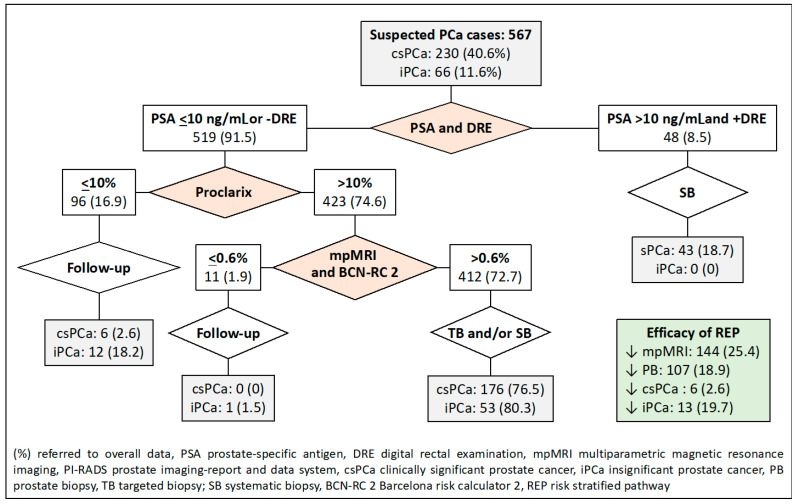
Flowchart and efficacy of the RSP-5, based on sequential stratifications PSA-DRE, Procla-rix™, and BCN-RC 2. Green is overall results.

The fifth RSP, based on PSA-DRE, Proclarix™, and BCN-RC 2 sequenced stratifications, is presented in Figure 6. MRI demand was reduced in 144 cases (25.4%), prostate biopsies in 107 cases (18.9%), and over-detection of iPCa in 13 cases (19.7%). Misdiagnosis of csPCa remained in six cases (2.6%).

**Figure 7:**
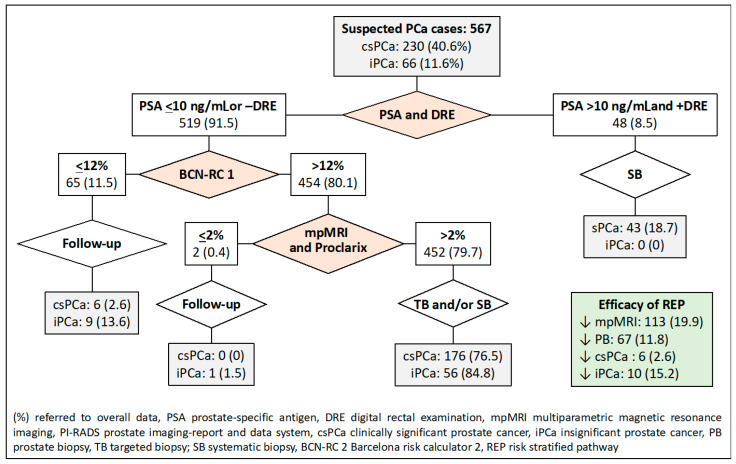
Flowchart and efficacy of the RSP-6, based on sequential stratifications from PSA-DRE, BCN-RC 1, and Proclarix™. Green is overall results.

The sixth RSP was based on sequential stratifications from PSA-DRE, BCN-RC 1, and Proclarix™, as shown in Figure 7. We note that 113 MRI scans (19.9%) would be avoided, as well as 67 prostate biopsies (11.8%) and 10 over-detected iPCa. Misdiagnosis of csPCa remained in six cases at 2.6%.

**Figure 8:**
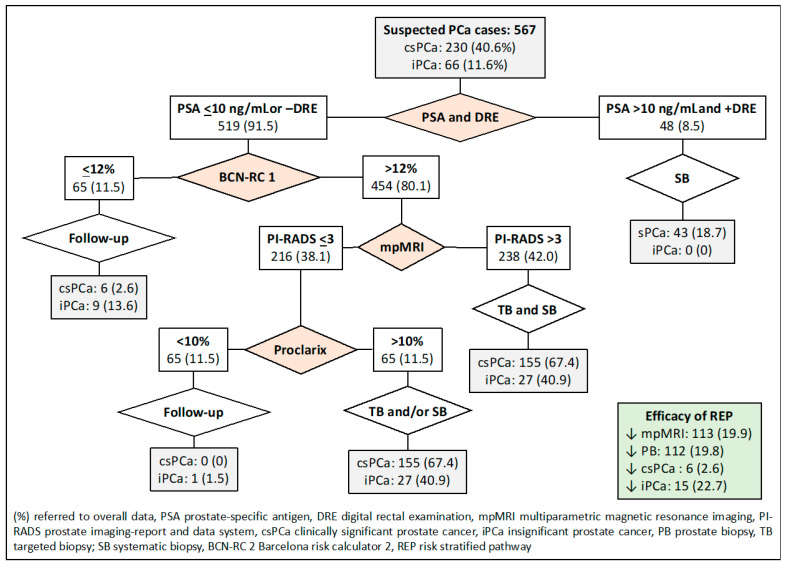
Flowchart and efficacy of the RSP-7, based on sequential stratifications from PSA-DRE, BCN-RC 1, mpMRI, and Proclarix™. Green is overall results.

Finally, the seventh RSP analyzed was based on sequential stratifications from PSA-DRE, BCN-RC 1, MRI, and Proclarix™, as shown in Figure 8. MRI demand was reduced in 113 individuals (19.9%), prostate biopsies in 112 (19.8%), and over-detection of iPCa in 15 (22.7%). Misdiagnosis of csPCa remained in six men (2.6%).

### 3.4. Efficacy of Proposed RSPs

The clinical efficacy of the proposed RSPs, based on the avoidance of mpMRIs and prostate biopsies and the reduction in the over-detection of iPCa, is summarized in Table 2. We note that the 2.6% rate of undetected csPCa in the currently recommended RSP [33] was fixed for all RSPs to facilitate comparisons between them. The decrease in demand for mpMRI ranged from 8.5 to 25.4%, while the decrease in prostate biopsies ranged from 12.9% to 19.8%. The decrease in over-detection of iPCa ranged from 13.6 to 22.7%. Selec-ting the most efficient RSP is challenging since there are three endpoint variables for comparison. However, if we consider the decrease in prostate biopsies as the most relevant outcome variable, only RSP-5 and -7 reduced prostate biopsies regarding the currently recommended RSP, in which 17.6% of prostate biopsies were avoided compared to 18.9% (*p* < 0.001) and 19.8% (*p* < 0.001), respectively. Significant differences were also found between RSP-5 and -7 (*p* < 0.001).

### 3.5. Cost-Effectiveness Approximative Analysis

Since four of the proposed RSPs included stratifications with Proclarix™ at different times, there was additional cost. We conducted a simple and limited cost-effectiveness approximation, evaluating savings according to the avoided costs of MRIs and prostate biopsies, which are EUR 280 and EUR 1200 each, respectively, as shown in Table 3. The RSP with sequencing stratifications based on PSA-DRE, BCN-RC 1, mpMRI, and Procla-rix™ was the most cost-effective while assuming that the price of Proclarix™ remained at less than EUR 200. The cost-effectiveness was similar to that of the recommended RSP when the price of Proclarix was EUR 200. 

## 4. Discussion

The present study aimed to contribute to the efforts of the EAU in promoting efficient screening for csPCa by proposing efficient RSPs. The goal is to reduce demand for MRI, unnecessary prostate biopsies, and the over-detection of iPCa. We note that all men suspected of having PCa in our study cohort underwent mpMRI and either targeted and systematic biopsies or systematic biopsies alone when the PI-RADS score was less than 3. The first proposed RSP aligns with the recommendation outlined in the EAU PCa guidelines [37]. MpMRI was always performed, while prostate biopsy was avoided in men with a PI-RADS score of less than 3. This approach resulted in a 17.6% reduction in prostate biopsies and a 13.6% reduction in iPCa over-detection. The observed 2.6% misdiagnosis for csPCa was fixed in the study to facilitate comparisons with proposed RSPs. This misdiagnosis in PI-RADS scores of less than 3 remains in the range from 2 to 4% (95% CI 0–9) reported with PI-RADS versions 2.1 [38] and 2.0 [39,40].

A proposed tool to be incorporated for the first stratification of men suspected of having PCa is based on a serum PSA level higher than 10 ng/mL and a suspicious DRE. We previously observed that mpMRI scanning can be avoided since targeted biopsies did not detect additional csPCa diagnosed in systematic biopsies in these men [27]. This tool has been used previously in stratifications carried out with BCN-RCs 1 and 2 in a previous RSP [28]. In the current study cohort, men with a serum PSA level above 10 ng/mL and suspicious DRE represented 8.5% of all men suspected of having PCa. This frequency seems higher than that observed in screening series, especially in secondary rounds [41,42]. Following MRI scanning in men with a serum PSA level of 10 ng/mL or less and normal DRE, a 2.6% misdiagnosis of csPCa was observed. In summary, this RSP avoided 8.5% of mpMRIs and 16.9% of prostate biopsies, and the over-detection of iPCa decreased by 13.6%.

In our third proposed RSP, a second stratification with Proclarix™, followed by PSA-DRE, was introduced. Men with Proclarix™ scores of less than 10% would undergo follow-up with a 2.6% csPCa misdiagnosis. Men with Proclarix™ levels above 10% would undergo mpMRI and targeted and/or systematic biopsies. This RSP reduced MRI demand by 25.4%, prostate biopsies by 16.9%, and iPCa over-detection by 18.2%. Proclarix™ was higher than 10% in all men with a serum PSA level above 10 ng/mL and suspicious DRE [23].

The fourth proposed RSP has been previously assayed. It stratifies men suspected of having PCa into PSA-DRE, BCN-RC 1, and BCN-RC 2. The BCN-RC 1 threshold was selected for obtaining a 2.6% csPCa misdiagnosis. In summary, this RSP decreased demand for mpMRI in 19.9% of cases, prostate biopsies in 12.9%, and the over-detection of iPCa in 15.2%. The same RSP has exhibited better results in a study of 946 men suspected of ha-ving PCa that used different thresholds for stratifications [27]. Rammers et al. have recently reported the results with an RSP based on sequencing the Rotterdam RC3 and the Rotterdam MRI-RC in the MRI arm of the PRECISION trial. After recalibration and adjustment of csPCa thresholds, 13% of mpMRI exams and 9% of prostate biopsies were avoided, missing 8.5% of csPCa detected in the 134 men with a PI-RADS score of ≥−3 in whom MRI-targeted biopsies of suspicious lesions and systematic biopsies were performed [28].

The last three proposed RSPs sequenced stratifications from Proclarix™ at different times. The fifth RSP stratifies men suspected of having PCa from PSA-DRE, Proclarix™, and BCN-RC 2. Demand for mpMRI decreased in 25.4% of cases, prostate biopsies in 18.9%, and the over-detection of iPCa in 19.7%, with a 2.6% misdiagnosis for csPCa. The sixth RSP sequenced stratifications from PSA-DRE, BCN-RC 1, and Proclarix™. MpMRI demand decreased in 19.9% of cases, prostate biopsies in 11.8%, and over-detection of iPCa in 15.2%. Finally, the seventh proposed RSP sequenced four stratifications in men suspected of having PCa according to PSA-DRE, BCN-RC 1, PI-RADS score, and Procla-rix™. Demand for mpMRI decreased in 19.9% of cases, prostate biopsies in 19.8%, and the over-detection of iPCa in 22.7%, with misdiagnosis of csPCa in 2.6%. This RSP exhibited the highest rate of avoided prostate biopsies and the highest decrease in the over-detection of iPCa. Due to its effectiveness, the seventh proposed RSP exhibited the highest total savings of all the proposed RSPs. Proclarix™ sequenced the fourth stratification in men with a PI-RADS score of 3 or lower, in whom its high sensitivity to csPCa has been previously described [24]. This RSP maintained the best cost-effectiveness when the price of ProclarixTM remained below EUR 200.

Currently, few RSP sequencing predictive models for stratification of men with suspected PCa have been reported [27,28]. The integration of modern markers with other tools as predictive models has been previously reported [17,18,43,44]. Modern RSPs should aim to stratify men suspected of having PCa in a cost-effective way.

The main limitation of our study is the difficulty in identifying the best RSP. The proposed RSPs are only part of the possible combinations using the four tools for sequencing stratifications of men suspected of having PCa and using different threshold combinations. The csPCa definition in prostate biopsies does not represent the true pathology in the entire prostate gland. The prices of mpMRI, prostate biopsy, and Procla-rix™ are not uniform across countries. Savings secondary to a decrease in iPCa over-detection have not been computed.

We believe that tools for stratifying men suspected of having PCa are available to design the most appropriate RSP at each site where screening for csPCa will be implemented. Future studies in the context of EAU PRAISE-U can report appropriate RSPs to implement csPCa screening across the EU. However, each proposed RSP must be validated for use at each site.

## 5. Conclusions

RSPs can improve the early detection of csPCa by reducing demand for MRI exams, prostate biopsies, and the over-detection of iPCa. There are several options to design efficient RSPs, and those stratifying men suspected of having PCa with several tools seem to be more efficient than those using only predictive models. Our most effective RSPs used sequential stratifications with PSA-DRE, BCN-RC 1, PI-RADS score, and Proclarix™. The cost-effectiveness of RSPs using modern tumor markers must be investigated.

## Figures and Tables

**Table 1:** Characteristics of the study cohort.

Characteristic	Measurement
Number of cases	567
Median age, years (IQR)	69 (63–74)
Median total PSA, ng/mL (IQR)	7.0 (4.9–11.2)
Abnormal DRE, *n* (%)	109 (19.2)
Median free PSA, ng/mL (IQR)	1.1 (0.7–1.7)
Median prostate volume, ml (IQR)	55 (40–76)
Median percent free PSA, % (IQR)	15.1 (10.7–20.6)
Median PSA density, ng/mL/cc (IQR)	0.13 (0.09–0.21)
Repeat biopsy, *n* (%)	133 (23.5)
Family history of PCa, *n* (%)	48 (8.6%)
PI-RADS, *n* (%)	
1–2	100 (17.6)
3	169 (29.8)
4	190 (33.5)
5	108 (19.0)
Overall PCa detection, *n* (%)	296 (52.2)
csPCa detection, *n* (%)	230 (40.6)
iPCa detection, *n* (%)	66 (11.6)

IQR—interquartile range; n—number; PCa—prostate cancer; csPCa—clinically significant PCa; iPCa—insignificant PCa.

**Table 2:** Effectiveness of risk-stratified pathways (REP), based on: (1) MRI; (2) serum PSA-DRE and MRI; (3) serum PSA-DRE and Proclarix™; (4) serum PSA-DRE, BCN-RC 1 and BCN-RC 2; (5) serum PSA-DRE, Proclarix™, and BCN-RC 2; (6) PSA-DRE, BCN-RC 1, and Proclarix™; (7) PSA-DRE, BCN-RC 1, MRI, and Proclarix™.

Pathway According to Stratifications	Thresholds	mpMRIs, n (%)	Prostate Biopsies, n (%)	csPCa Detection, n (%)	Over-Detection iPCa, n (%)
1. mpMRI (currently recommended)	PI-RADS 2	0 (0)	100 (17.6)	6 (2.6)	9 (13.6)
2. PSA-DRE, and mpMRI	PSA 10 and +DRE; PI-RADS 2	48 (8.5)	96 (16.9)	6 (2.6)	9 (13.6)
3. PSA-DRE, Proclarix™	PSA 10 and +DRE; Proclarix 10	144 (25.4)	96 (16.9)	6 (2.6)	12 (18.2)
4. PSA-DRE, BCN-RC1, and BCN-RC2	PSA 10 and +DRE, BCN-RC1 12, BCN-RC2 0.7	113 (19.9)	73 (12.9)	6 (2.6)	10 (15.2)
5. PSA-DRE, Proclarix™, and BCN-RC2	PSA 10 and +DRE, Proclarix 10, BCN-RC2 0.6	144 (25.4)	107 (18.9)	6 (2.6)	13 (19.7)
6. PSA-DRE, BCN-RC1, and Proclarix™	PSA 10 and +DRE, BCN-RC1 12, Proclarix 10	113 (19.9)	67 (11.8)	6 (2.6)	10 (15.2)
7. PSA-DRE, BCN-RC1, mpMRI, and Proclarix™	PSA 10 and +DRE, BCN-RC1 12, PI-RADS 3, Proclarix 10	113 (19.9)	112 (19.8)	6 (2.6)	15 (22.7)

mpMRI—multiparametric magnetic resonance imaging; PI-RADS—prostate imaging–reporting and data system; csPCa—significant prostate cancer; iPCa—insignificant PCa; n—number; PSA—prostate-specific antigen; DRE—digital rectal examination; BCN-RC—Barcelona-risk calculator.

**Table 3:** Approximation to the cost-effectiveness, savings valuated in euros, of risk-stratified pathways (REP), based on: (1) MRI; (2) Serum PSA-DRE and MRI; (3) Serum PSA-DRE and Proclarix™; (4) Serum PSA-DRE, BCN-RC 1 and BCN-RC 2; (5) Serum PSA-DRE, Proclarix™, and BCN-RC 2; (6) PSA-DRE, BCN-RC 1, and Proclarix™; (7) PSA-DRE, BCN-RC 1, MRI, and Proclarix™.

Pathway According to Stratifications	Avoided mpMRIs	mpMRI Savings	Avoided Prostate Biopsies	Prostate Biopsy Savings	MRI and Prostate Biopsy Savings	Proclarix™ Use	Proclarix™ Cost	Total Savings
1. mpMRI (currently recommended)	0	0	100	120,000	120,000	0	0	120,000
2. PSA-DRE, and mpMRI	48	13,440	96	115,200	128,400	0	0	128,640
3. PSA-DRE, Proclarix™	144	40,320	96	115,200	155,520	519	103,800	51,900
4. PSA-DRE, BCN-RC1, and BCN-RC2	113	31,840	73	37,600	119,240	0	0	119,240
5. PSA-DRE, Proclarix™, and BCN-RC2	144	40,320	107	128,400	168,720	519	103.800	64.920
6. PSA-DRE, BCN-RC1, and Proclarix™	113	31,640	67	80,400	112,040	454	90,800	21,240
7. PSA-DRE, BCN-RC1, mpMRI, and Proclarix™	113	31,640	112	134,400	166,040	216	43,200	122,840

mpMRI—multiparametric magnetic resonance imaging; PI-RADS—prostate imaging–reporting and data system; csPCa—significant prostate cancer; iPCa—insignificant PCa; n—number; PSA—prostate-specific antigen; DRE—digital rectal examination; BCN-RC—Barcelona-risk calculator.

## Data Availability

The data presented in this study are available on request from the corresponding author.
